# The Standard of Care Definitions on COVID-19 Pharmacological Clinical Trials: A Systematic Review

**DOI:** 10.3389/fphar.2021.749514

**Published:** 2021-10-18

**Authors:** Antonio Addis, Laura Amato, Fabio Cruciani, Rosella Saulle, Franco De Crescenzo, Zuzana Mitrova, Simona Vecchi, Francesco Perrone, Marina Davoli

**Affiliations:** ^1^ Department of Epidemiology, Lazio Regional Health Service, Rome, Italy; ^2^ Department of Psychiatry, Warneford Hospital, University of Oxford, Oxford, United Kingdom; ^3^ Clinical Trial Unit, Istituto Nazionale Tumori IRCCS Fondazione Pascale, Napoli, Italy

**Keywords:** standard of care (SOC), COVID–19, randomized clinical trial (RCT), randomized clinical trial design, drug treatment

## Abstract

**Background:** Standard of Care (SoC) has been used with different significance across Randomized Clinical Trials (RCTs) on the treatment of Covid-19. In the context of a living systematic review on pharmacological interventions for COVID-19, we assessed the characteristics of the SoC adopted in the published RCTs.

**Methods:** We performed a systematic review searching Medline, Pubmed, Embase, Cochrane Covid-19 register, international trial registers, medRxiv, bioRxiv, and arXiv up to April 10, 2021. We included all RCTs comparing any pharmacological intervention for Covid-19 against any drugs, placebo, or SoC. All trials selected have been classified as studies with SoC including treatments under investigation for COVID-19 (SoC+); studies with SoC without specifications regarding the potential therapies allowed (SoC-); studies including as control groups Placebo (P) or active controls (A+).

**Results:** We included in our analysis 144 RCTs, comprising 78,319 patients. Most of these trials included SoC (108; 75.0%); some in all arms of the study (69.7%) or just as independent comparators (30.3%). Treatments under investigation for COVID-19 in other trials were included in the SoC (SoC+) in 67 cases (62.0%), Thirty-one different therapeutic agents (alone or in combination) were counted within the studies with SoC+: mostly hydroxychloroquine or chloroquine (28), lopinavir/ritonavir (20) or azithromycin (16). No specification was given regarding treatment allowed in the control groups (SoC-) in 41 studies (38.0%).

**Conclusion:** Our analysis shows that the findings emerging from several clinical trials regarding the efficacy and safety of pharmacological intervention for COVID-19 might be jeopardized by the quality of control arms.

## Introduction

The Council for International Organizations of Medical Sciences guidelines state that participants in the control group of a trial must receive an established effective intervention. In this context, a degree of consensus and acceptability among health professionals about the nature of the standard of care (SoC) is identified by using the term “established effective therapy” (Council for International, 2016). In the past, the correct interpretation of the SoC has generated a lot of controversies among researchers (Bhutta, 2004) general practitioners (Moffett and Moore, 2011), and policy makers (Collins et al., 2020). SARS-COV 2 (severe acute respiratory syndrome coronavirus 2) pandemic fuelled a great number of clinical trials on different types of interventions to treat COVID-19 (Herper, 2020). Most of these planned interventions are measuring efficacy in comparison with a SoC or other comparators even though a definition of best standard treatment was not available. One potential concern with these studies is that with the effort to respond quickly to the COVID-19 pandemic emergency obtaining favourable results, the SoC of these studies could have been not well defined. Using suboptimal SoC may bias a trial in favor or even against the experimental arm and reduces external validity–i.e. the trial is no longer capable of answering the potential clinical question of whether the proposed new agent against COVID-19 is effective and safe. Despite concern for undefined SoC in COVID-19 studies, to our knowledge, there is no empirical analysis assessing characteristics of the treatments allowed within the SoC definition. For these reasons, we analyzed the characteristics of SoC adopted in the COVID-19 pharmacological clinical trials.

## Methods

This study is part of a living review of pharmacological agents for the treatment of Covid-19 conducted by the Department of Epidemiology of the Regional Health Service Lazio, Italy, to inform national regulatory agencies and clinicians, and available at https://www.deplazio.net/farmacicovid. This living review was recently published in a paper and employs both pair-wise meta-analysis and network meta-analysis methods in order to synthesize and assess the comparative efficacy and safety of these drugs (Moffett and Moore, 2011; Collins et al., 2020). This activity is also part of the rolling collaborative reviews published monthly with the European Network of Health Technology Assessment (EUnetHTA) and available at https://eunethta.eu/covid-19-treatment/.

### Search Strategy and Selection Criteria

We searched Medline, PubMed, and Embase from December 2019 to April 10, 2021 (for details see Supplementary Appendix). We searched medRxiv.org (https://www.medrxiv.org/), bioRxiv.org (https://www.bioRxiv.org/), andarXiv.org (https://www.arXiv.org/) for preprints of preliminary reports of randomized trials. We also searched the CochraneCovid-19 Study Register (https://covid-19.cochrane.org/), ClinicalTrials.gov (www.clinicaltrials.gov) and World Health Organization (WHO) International Clinical Trials Registry Platform (ICTRP) (www.who.int/ictrp/en/). Since March 2021 we have also searched the L.OVE platform (https://app.iloveevidence.com/loves/5e6fdb9669c00e4ac072701d?utm=aile). Additional sources included journal alerts, contact with researchers, websites such as Imperial College, London School of Hygiene and Tropical Medicine, and Eurosurveillance. We applied no restriction on the language of publication. We included parallel randomized clinical trials (RCTs) comparing any pharmacological intervention against another pharmacological intervention or placebo or standard care (SC), for the treatment of individuals with Covid-19. There were no limits in terms of gender or ethnicity or severity of the disease. We included pharmacological interventions without restrictions on dosage, regimen, dosing interval, route of administration, or intervention duration. Since March 1, 2021, we decided to include in our synthesis only those trials with equal or more than 100 individuals randomized. We included standard care as defined by study authors. Most studies had the standard of care (SoC) or control arms, and all trials selected have been classified according to the following definitions: 1. Studies with SoC including treatments under investigation for COVID-19 (SoC+); 2. Studies with SoC without specifications regarding the potential therapies allowed in the control groups (SoC-); 3. Studies including Placebo in the control group (P); and finally, 4. Studies including in the control group one or more active control (A+). We did not include quasi-randomized controlled trials, cross-over trials, or pilot studies with a single arm. We excluded studies comparing two dosages of the same pharmacological agent.

We did not exclude studies on individuals with a comorbid disorder.

#### Data Extraction

Four authors (LA, FC, RS, SV) independently screened the references retrieved by the search, selected the studies, and extracted the data, using a predefined data-extraction sheet, including the following data: First author or acronym; year of publication; study design; participants; diagnosis, sample size, mean age, gender distribution, the severity of illness, setting; interventions; comparators; details regarding the SoC when available; the number of patients allocated to each arm, drug name, dose, duration of the interventions and follow-up.

#### Data Synthesis

Because the purpose of this study was to describe the characteristics of SoC adopted in the COVID-19 pharmacological clinical trials, these were summarized narratively. We did not perform a quantitative synthesis of the studies because this was not the focus of the review.

## Results

**FIGURE 1:**
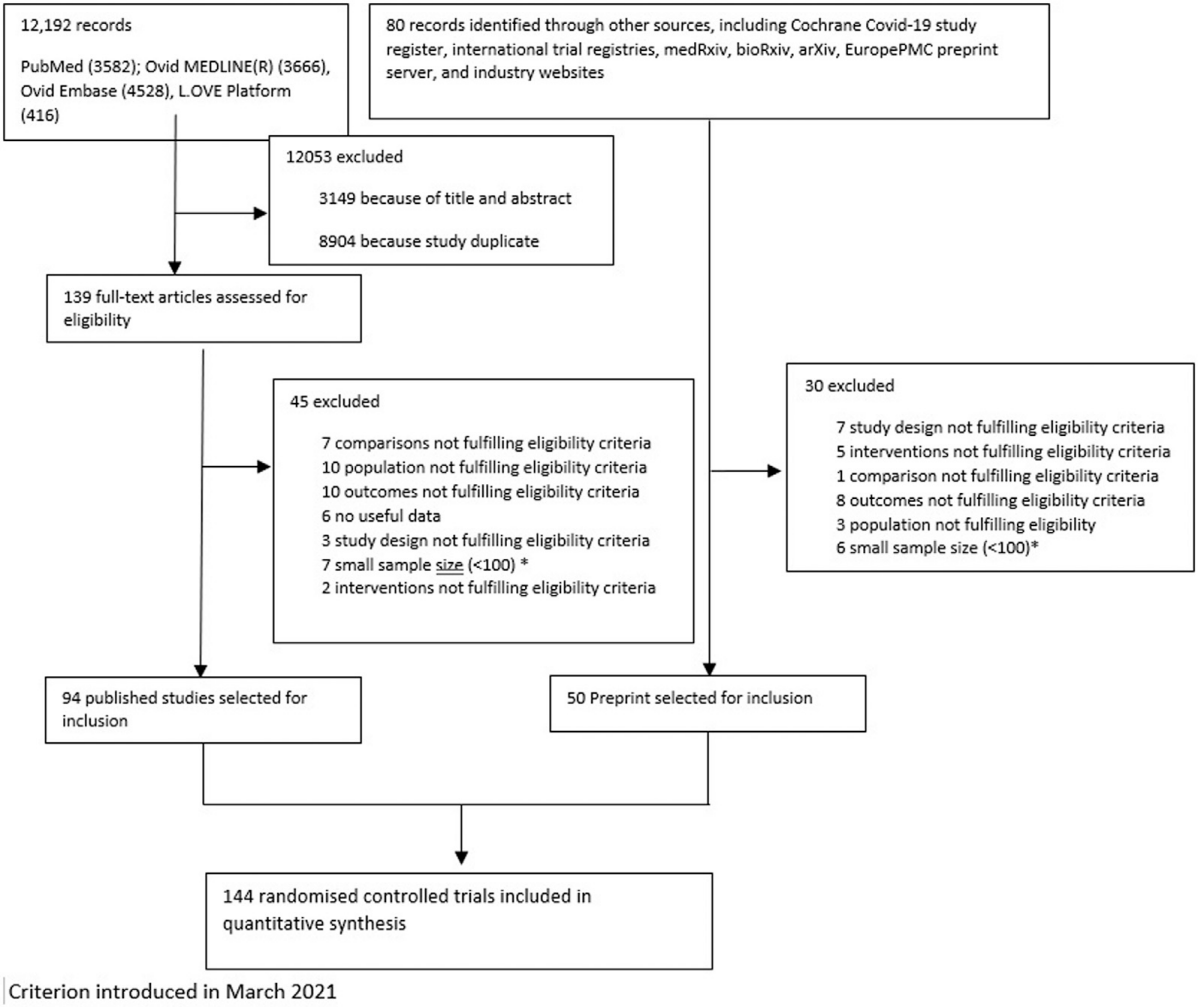
Flow chart reporting the results of the search strategy.

We identified 12,192 citations and included 139 articles that were evaluated in full-text; from these 94 peer-reviewed publications were selected. In addition, 50 preprint articles were identified through other sources including Cochrane Covid-19 study register, international trial registries, medRxiv, bioRxiv, arXiv, EuropePMC preprint server, and industry websites. Following selected exclusion, as reported in Figure 1, a total of 144 trials were included, which randomized 78,319 patients to 81 pharmacological treatments or a combination of treatments or SoC or placebo. Summary of the characteristics of included studies, and a full list of references is available in Supplementary Appendix.

**FIGURE 2:**
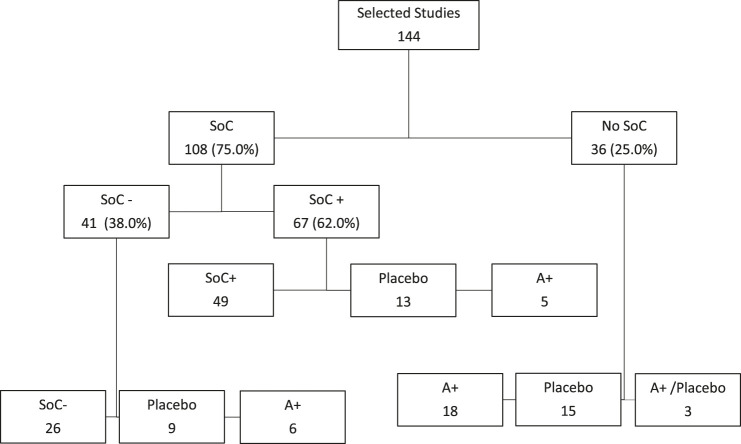
Inclusion or exclusion of Standard of Care (SoC) in the selected studies as comparators. 1. **SoC+** = Studies with Standard of Care including treatments under investigation for COVID-19; **SoC−** = Studies with Standard of Care without specifications regarding the potential therapies allowed in the control groups; **Placebo** = Studies with Placebo in the control group; **A+** = Studies including in the control group one or more active control.

The mean study sample size was 515.3 participants (SD 1246.7). In total, 37,704 participants were randomly assigned to an active drug or combination of active drugs, and 40,615 were randomly assigned to a placebo or SoC. The mean age was 53.4°years (SD 9.3), while less than half (40.7%) of the sample population were women. The average duration of the treatment in the studies was 10°days (SD 6.5); in 4 studies treatment duration was not reported and in 10 studies administration of treatment was in one single infusion or 1 hour of infusion. The average duration of follow-up was 28°days (SD 21.3), 19 studies did not report duration of follow-up and in two studies follow-up was until discharge from hospital. Figure 2 shows how most of the studies on COVID 19 compared any kind of potential agent against COVID 19 with SoC (108, 75.0%), while 36 (25.0%) studies did not use any kind of SoC. When the SoC was adopted, it was used in all arms of the study (69.7%) or just as a single comparator (30.3%). In studies without SoC, the efficacy of new proposed therapies was measured only versus other active drugs (A+ = 18), Placebo (*p* = 15), or both (A+/P = 3). Studies using SoC often included treatments under investigation for COVID-19 in other trials (SoC+ = 67; 62.0%), otherwise, there is no specification regarding the potential therapies allowed in the control groups including SoC (SoC- = 41; 38.0%). Out of all selected studies with SoC, Placebo or Active control (A+) were used as comparators in 22 (20.4%) and 11 (10.2%) studies, respectively.

Table 1 shows all 31 different therapeutic agents (alone or in combination) described in the published report in the section reporting the details of the SoC. These included treatments under investigation for COVID-19 in other trials (SoC+). According to these data antivirals, antibiotics, glucocorticoids, anticoagulants, and aminoquinolines were often allowed as SoC. In particular, hydroxychloroquine or chloroquine (28 studies), lopinavir/ritonavir (20 studies), or azithromycin (16 studies) were included as potential treatments allowed as SoC.

**TABLE 1:** Active agents allowed in the studies with standard of care including treatments under investigation for COVID-19 (SoC+).

Class of drugs	Drugs	No. of studies
Aminoquinolines	hydroxychloroquine	24
	Chloroquine	3
Antiviral	Oseltamivir	7
	Remdesivir	5
	ribavirin	3
	Lopinavir/ritonavir	20
	Atazanavir/ritonavir	2
	Umifenovir	4
	Darunavir/cobicysteate	1
	Lopinavir	2
	Others, unspecified	19
Antibiotics	nitazoxanide	3
	azithromycin	16
	Ceftriaxone	2
	levofloxacin	1
	moxifloxacin	1
	doxycycline	1
	others unspecified	27
Glucocorticoids	hydrocortisone	1
	others unspecified	26
Vitamins And Minerals	vitamin C	4
	vitamin D	4
	zinc	2
Immunosuppressant	Tocilizumab	8
	anakinra	1
	plasma Conv	4
Immunostimulants	interferon alpha	4
	interferon beta	1
Anticoagulants	Heparin	9
	others unspecified	5
NSAID	naproxen	1
	others unspecified	2
Others Therapies	paracetamol	7
	Oxygen	20
	ivermectin	1
	antifungal drugs	1
	Chinese herbal therapy	4

## Discussion

In medicine the RCT design remains the gold standard methodology to test the efficacy of new drug treatments, even when the level of uncertainty is very high. However, how can we measure the superiority of new agents versus the best standard of care when such “standard” still does not exist? An emergency like a pandemic event is, by definition, associated with an undefined usual care. In this context, it is not a surprise to find that many ongoing trials were designed in absence of a clear definition for the best SoC in COVID-19. Nevertheless, according to our analysis, taking into account all selected studies, several authors were not hesitant to compare the new agents with experimental treatments as active controls (35; 24.3%) or within the so-called SoC (67; 46.5%). Furthermore, even though the choice of the comparator is crucial to understand the real efficacy and safety of new drug treatment, several studies do not report enough information on the characteristics of the SoC (36; 25.0%). In principle, lack of knowledge in the SoC might not significantly bias the comparison if the experimental drug is a pure add-on therapy and SoC is the same in both arms However, while lack of knowledge in the SoC does not significantly bias the comparison if the experimental drug is a pure add-on therapy and SoC is the same in both arms, we found that in 30.3% of all selected studies the comparison was head-to-head between the new drug and SoC. In this case, risk of bias still remains high for at least two reasons: first, a “non-evidence-based” SoC may increases (or decreases) the outcome probabilities in both arms interfering with the study power to find out a difference; and, second, a “non-evidence-based” SoC may produces pharmacologic interference with the experimental drug and it can bias safety and efficacy results.

On the other side, non-evidence-based control arms severely undermine the value of comparisons of experimental drugs vs. SoC, because comparing two treatments each with unknown efficacy produces results that cannot be interpreted, even in presence of a statistically significant difference in favor of one.

As soon as the global SARS-CoV-2 pandemic became more critical, the scientific community responded launching an increasing number of studies (Bhutta, 2004). Clinicians looked for medicine ready to use, no matter the level of evidence available and this pushed researchers to plan studies and write protocols in a very short time. In this context, national competent authorities might experience difficulties in the promotion of good clinical trials to manage a rapidly evolving emergency (Collins et al., 2020; Herper, 2020).

The clinical trials aiming to verify the safety and efficacy of different treatments or interventions for COVID 19 may not be very well established, due to the complexity of the context in which they are performed.By the other hand, the high number of trials and of the included patients may overcome this limitation. Evidence about possible treatments changed almost on daily or weekly bases during the investigation period of the study, therefore this can influence on making confusion and having conflicting evidence.

Our analysis could be also limited by the kind of information allowed in the publications reporting clinical trial results that we used as a source of information. These data may be more precise and detailed in the full protocols. However, this does not have an impact on the large number of studies we identified comparing experimental intervention for COVID-19 to a control or a SoC including treatments still under investigation for COVID-19 in other studies. Our analysis is not capable to measure how the SoC changed on time because it is not easy to time frame all different studies according to different pandemic periods taking into account design of the study and the time of the publication. However, it is possible that the definition and reporting of SoC became more accurate over time.

At the best of our knowledge there was not a general consensus, shared by international regulatory bodies (FDA, EMA, WHO, etc), regarding the common definition of the SoC in the COVID 19 emergency. A recent study on institutional, open-source COVID 19 guidelines showed large variability in treatment or supplemental pharmacological recommendations (Collins et al., 2020).

In conclusion, this analysis shows that the findings of several ongoing clinical studies regarding the efficacy and safety of pharmacological intervention for COVID-19 ARE potentially jeopardized. Data on efficacy and safety of new treatments, produced in a rapidly evolving emergency, without a clear definition of SoC, are potentially exposed to a higher risk of bias (Addis et al., 2020; Ledford, 2020; Cruciani et al., 2021; De Crescenzo et al., 2021).
