# Supplementary material for: The Standard of Care Definitions on COVID-19 Pharmacological Clinical Trials: A Systematic Review
Source: Front Pharmacol. 2021 Oct 18;12:749514. doi: 10.3389/fphar.2021.749514 (PMC8558371; doi:10.3389/fphar.2021.749514)
Supplement: Supplementary file 1 [file DataSheet1.pdf]

Supplementary Table 1. Studies included in the analysis

	Study<br>(First Name)	Country	N	Intervention<br>(N of patients)	Comparisons<br>(N of Patients)	*Details on agents included (or excluded) in the Standard of Care*	CODE	Participants
1.	Abbaspour Kasgari 2020	IR	48	1. Sofosbuvir + Daclatasvir + Ribavirin N=24;	2. SoC N=24	HydroxyChl + Lop/Rit, with or without ribavirin	SoC+	NR
2.	Abd-Elsalam 2020a	EG	194	1. HydroxyChl +SoC N=97;	2. SoC N=97	Paracetamol, O2, fluids, antibiotic (cephalosporins), oseltamivir, and invasive mechanical ventilation with Hydrocort for severe cases	SoC+	severe
3.	Abd-Elsalam 2020b	EG	191	1. HydroxyChl +Zinco+ SoC N=96;	2.SoC N=95	No details on SoC reported	SoC-	moderate, severe
4.	ACTIV-3/TICO	USA, DK, SGP	326	1. LY-CoV555 +SoC N= 169;	2. Placebo + SoC N= 157	Remdes, O2 and glucocorticoids	SoC+; P	NR
5.	Ader 2021	FR	604	1. Lop/Rit N=150;<br>2. Lop/Rit + interferon beta 1a N= 150;	3. HydroxyChl N=151;<br>4. SoC N=152	No details on SoC reported	SoC-; A+	moderate, severe
6.	Agarwal 2020 (PLACID trial)	IN	464	1. Plasma C +SoC N=235;	2. SoC N=229	HydroxyChl, Remdes, Lop/Rit, Oseltamivir, broad-spectrum antibiotics, steroids, Tocilizumab and supportive therapy (O2 via nasal cannula, face mask, non-respiratory face mask; non-invasive or invasive mechanical ventilation	SoC+	moderate

7.	Ahmed 2020	BD	72	1. Ivermectin N=24;<br>2. Ivermectin + Doxycycline N=24;	3. Placebo N=24	Placebo	P	NR
8.	Alavi Darazam 2020	IR	60	1. Interferon beta 1 a N=20;	2. Interferon beta 1 b N=20;<br>3. SoC	Lop/Rit +HydroxyChl (single dose)	SoC+	moderate
9.	AlQahtani 2020	BRN	40	1. Plasma C N=20;	2. SoC N=20	Paracetamol, antivirals, tocilizumab, antibacterials	SoC+	moderate
10.	Ansarin 2020	IR	78	1. Bromesine hydrochloride N=39;	2. SoC N=39	All patients received national COVID-19 treatment protocol and best practice guidelines and also the "HydroxyChl 200 mg/d for two weeks" in addition to supportive and symptomatic therapy.	SoC+	moderate
11.	Avendaño-Solà 2020	E	81	1. Plasma C + SoC N=38;	2. SoC N=43	No details on SoC reported	SoC-	moderate
12.	Babalola 2021	NG	62	1. Ivermectina (6mg) N=21;<br>2. Ivermectina (12mg) N=21;	3. Lop/Rit N=20	No SoC	A+	NR
13.	Bajpai 2020	IN	31	1. Plasma C N= 15;	2. Fresh frozen plasma N= 16	No SoC	A+	Severe
14.	Balykova 2020	RUS	200	1. Favipiravir N=100;	2. SoC N=100	No details on SoC reported	SoC-	moderate
15.	Beigel 2020	USA, DK, UK, GR, DE, KR, MEX, ES, JP SGP	1062	1. Remdes + SoC N=541;	Placebo + SoC N=521	No details on SoC reported	SoC-; P	severe, moderate
16.	Blum 2021	BR	50	1. Nitazoxanide N=25;	2. Placebo N=25	NO SoC	P	moderate
17.	Brown 2020	USA	85	1. HydroxyChl;	2. Azithro	NO SoC	A+	NR

18.	Cadegiani 2020	BR	214	1. Proxalutamide +SoC N=114;	2. Placebo + SoC N=100	Nitazoxanide, Azithro	SoC+; P	moderate
19.	Cadegiani 2021 a	BR	130	1. Dutasteride + SoC N=64;	2. Placebo+ SoC N=66	Nitazoxanide, Azithro	SoC+; P	moderate
20.	Cadegiani 2021 b	BR	236	1. Proxalutamide + SoC N=171;	2. Placebo + SoC N=65	Nitazoxanide, Azithro	SoC+; P	moderate
21.	Cao B 2020	CN	199	1. Lop/Rit + SoC N=99;	2. SoC N=100	Supplemental O2, noninvasive and invasive ventilation, antibiotic agents, vasopressor support, renal-replacement therapy, and extracorporeal membrane O2ation	SoC+	severe
22.	Cao Y 2020	CN	43	1. Ruxolitinib + SoC N=22;	2. SoC N=21	Antiviral therapy, supplemental O2, non-invasive and invasive ventilation, corticosteroids, antibiotic agents, vasopressor support, renal replacement therapy and extracorporeal membrane O2ation (ECMO) +Placebo (100mg vitamin C)	SoC+; P	severe
23.	Castillo 2020	E	76	1. Calcifediol N=50;	2. SoC N=26	Hospital protocol: a combination of HydroxyChl and Azithro	SoC+	moderate/severe
24.	Cavalcanti 2020	BR	667	1. HydroxyChl + Azithro + SoC N=217; 2. HydroxyChl + SoC N=221;	3. SoC N=229	O2; antibiotics, corticosteroids, other immunomodulators (e.g., tocilizumab), other antivirals (e.g., oseltamivir for suspected influenza coinfection), as recommended by the attending physician.	SoC+	mild, moderate
25.	Chaccour 2021	USA	24	1. Ivermectin N=12;	2. Placebo N=12	No SoC	P	Mild

26.	Chen C 2020	CN	236	1. Favipiravir + SoC N=116;	2. Umifenovir + SoC N=120	Routine therapy but the details are No details on SoC reported. Other drugs were used for conventional therapy and symptomatic treatment to improve adverse reactions: Antibiotic, Antiviral drugs Glucocorticoid, Chinese herbal medicine, Psychotropic substances, Immunomodulator, Nutritional support	SoC+; A+	moderate/severe
27.	Chen CP 2020	TW	33	1. HydroxyChl N=21;	2. SoC N=12	Ceftriaxone; or levofloxacin or moxifloxacin for subjects allergic to ceftriaxone or Azithro or according to physician discretion. Oseltamivir will be administered for 5 days to subjects presenting with concomitant influenza A or B infection.	SoC+	Mild, moderate
28.	Chen J 2020 A	CN	30	1. HydroxyChl N=15;	2. HydroChloroq N=12;<br>3. SoC N=10	O2 therapy, and symptomatic supportive treatment. Viral drugs such as alpha interferon nebulization, oral Lop/Rit (clepivir), etc., and antibacterial drugs are given if necessary.	SoC+; A+	moderate
29.	Chen J 2020 B	CN	30	1. Darunavir/Cobicistat +SoC N=15;	2. SoC N=15	Interferon alpha 2b and the standards of care according to the recommendations of the guidelines in China	SoC-	moderate

30.	Chen L 2020	CN	67	1. Chloroq N=25;	2. HydroxyChl N=28;<br>3. SoC N=14	Chinese diagnosis and treatment protocol for new coronavirus pneumonia (5th edition)	SoC-; A+	moderate
31.	Chen Z 2020	CN	62	1. HydroxyChl + SoC N=31;	2. SoC N=31	O2 therapy, antiviral agents, antibacterial agents, and immunoglobulin, with or without corticosteroids	SoC+	moderate
32.	Cheng LL 2020	CN	200	1. Human Granulocyte Colony Stimulating Factor (rhG-CSF) + SoCN=100;	2. SoC N=100	Supplemental O2, non-invasive ventilation, or intravenous antibiotics when indicated	SoC-	moderate/severe
33.	Chowdhury 2020	BW	125	1. Ivermectin + Doxycycline N=63	2. HydroxyChl + Azithro N=62	No SC	A+	mild
34.	Chowdhury 2021	BW	606	1. Povidone iodine + symptomatic treatment N=303;	2. lukewarm water (placebo) + symptomatic treatment N=303	Placebo	P	mild
35.	CORIMUNO trial 2021	FR	116	1. Anakinra (interleukin 1 antagonist) +- SoC N=59;	2. SoC N=57	Usual care (antibiotic drugs, antiviral drugs, corticosteroids, vasopressor support, anticoagulants) was provided at the discretion of the site clinicians.	SoC-	mild / moderate, severe, critical
36.	Corral-Gudino 2020	E	63	1. Corticosteroid (Metylprednisolone)+ SoC N=34;	2. SoC N=29	Symptomatic treatment with paracetamol, O2 therapy, thrombosis prophylaxis with low molecular weight heparin and antibiotics for co-infections. Azythro, HydroxyChl and Lop/Rit	SoC+	severe
37.	Dabbous 2020	EG	100	1. Favipiravir N=50;	2. SoC N=50	HydroxyChl + oseltamivir	SoC+	mild / moderate

38.	Dabbous 2021	EG	96	1. Favipiravir N=44;	2. Chloroq N=48	No SoC	A+	mild / moderate
39.	Davoudi-Monfared 2020	IR	92	1. Interferon β-1a + SoC N=46;	2. SoC N=46	Hospital protocol: HydroxyChl + Lop/Rit or atazanavir/ritonavir for 7-10 days. Primary care, respiratory support, fluids, electrolytes, analgesics, antipyretics, corticosteroids and antibiotics have also been recommended in the hospital protocol if indicated.	SoC+	mild /severe
40.	Deftereos 2020_GRECCO Trial	GR	110	1. Colchicine N=56;	2. SoC N=54	No details on SoC reported	SoC-	NR
41.	Dequin 2020	FR	149	1. Corticosteroid (Hydrocortison) + SoC N=76;	2. Placebo + SoC N=73	No details on SoC reported	SoC-; P	Severe and critical
42.	Duarte 2020	RA	82	1. Telmisartan +SoC N=41;	2. SoC N=41	No details on SoC reported	SoC-	NR
43.	Dubée 2020	FR	250	1. HydroxyChl + SoC N=125;	2. Placebo + SoC N=125	Concomitant treatment with Azithro, Lop/Rit, treatment with corticosteroids	SoC+	Mild, moderate
44.	Edalatifard 2020	IR	68	1. Methylprednisolone + SoC N=34;	2. SoC N=34	HydroxyChl sulfate, Lopinavir, and Naproxen	SoC+	severe
45.	Esquivel-Moynelo 2020	C	79	1. Interferon alfa 2b + interferon gamma + Lop/Rit + Chloroq N=41;	2. Interferon alfa 2b + Lop/Rit + Chloroq N=38	No SC	A+	mild-to-moderate
46.	Feld 2020	CA	60	1. Perginterferon lambda N=30;	2. Placebo N=30	No SC	P	mild
47.	Furtado 2020	BR	447	1. HydroxyChl + Azithro N=237;	2. HydroxyChl N=210	No SC	A+	moderate/severe

48.	Galan 2021	E	168	1. HydroxyChl N=54;	2. Chloroq N=61;<br>3. Ivermectin N=53	No SC	A+	mild
49.	Gharbharan 2020	NL	86	1. Plasma C + SoC N=43;	2. SoC N=43	Depending on the hospital, off-label use of EMA-approved drugs (e.g., Chloroq, Azithro, Lop/Ritr, tocilizumab, anakinra) as treatment for COVID-19 was allowed in hospitals where this was part of the SC	SoC+	moderate/critical
50.	Ghandehari 2021	USA	42	1. Progesterone + SoC N=18;	2. SoC N=22	No details on SoC reported	SoC-	moderate
51.	Garebaghi 2020	IR	59	1. Immunoglobulin + SoC N=157;	2. Placebo + SoC N=155	All patients continued to receive initial treatmens including methods including at least both one antiviral and one Chloroq-class drug	SoC+	severe
52.	Gonzalez-Ochoa 2020	MEX	312	1. Sulodexide;	2. Placebo	No SoC	P	mild
53.	Gottlieb 2021	USA	592	1. LY-CoV555 700mg N=101;<br>2. LY-CoV555 2800mg N=107;<br>3. LY-CoV555 7000mg N=101;<br>4. LY-CoV555 2800mg+Etesevimab 2800 N=112;	5. Placebo N=156	No SoC	P	mild/moderate
54.	Guvenmez 2020	TR	24	1. Lincomycin N=12;	2. Azithro N=12	No SoC	A+	moderate

55.	Hashim 2020	IR	140	1. Ivermectina +Doxiciclina N=70;	2. SoC N=70	All or some of the following, according to the clinical condition of each patient: Acetaminophen; Vitamin C; Vitamin D3; Azithro; O2 therapy/ C-Pap if needed; Dexamethazone or methylprednisolone, if needed; Mechanical ventilation, if needed	SoC+	moderate/severe
56.	Hermine 2020	FR	131	1. Tocilizumab + SoC N=64;	2. SoC N=67	All patients received antibiotic agents, antiviral agents, corticosteroids, vasopressor support, anticoagulants	SoC+	moderate/critical
57.	Hernandez-Cardenas 2021	MEX	214	1. HydroxyChl + SoC N=108;	2. Placebo + SoC N=106	Clarithromycin or Azithro; carbapenem; Oseltamivir; Lop/Ritr;anticoagulants; corticosteroids (Methylprednisolone or Dexamethasone)	SoC+	moderate
58.	Ho 2021	USA	4313	1. Corticosteroids N=574;	2. SoC N=3739	adjuvant therapies, including tocilizumab and anticoagulants	SoC+	moderate/severe
59.	Huang 2020	CN	22	1. Chloroq N=10;	2. Lop/Rit N=12	No SoC	A+	moderate/severe
60.	Huang Y-Q 2020	CN	101	1. Ribavirin plus Interferon α N=33;	2. Ribavirin + Lop/Rit + Interferon α N=36;<br>3. Lop/Rit + Interferon α N=32	No SoC	A+	moderate
61.	Hung 2020	CN	127	1. Lop/Rit + ribavirin + interferone β -1b + SoC N=86;	2. Lop/Rit + SoC N=41	O2 therapy; non-invasive ventilation; supportive ventilation; antibiotics; corticosteroids.	SoC+; A+	moderate

62.	Ivashchenko 2020	RU	60	1. Favipiravir (1600/600 mg) N=20;<br>2. Favipiravir (1800/800 mg) N=20;	3. SoC N=20	No details on SoC reported	SoC-	moderate
63.	Jagannathan 2021	USA	120	1. Peginterferon Lambda-1a (180mcg in single dose) N=60;	2. Placebo N=60	No SoC	P	
64.	Jamaati 2021	IR	50	1. Dexamethasone +SoC N=25;	2. SoC N=25	O2 support, fluid support, Lop/Ritr	SoC+	moderate
65.	Jeronimo 2020	BR	416	1. Corticosteroid (Methylprednisolone) + SoC N=209;	2. Placebo + SoC N=207	All patients received a combination of ceftriaxone plus a macrolide. All patients meeting ARDS criteria used pre-emptively intravenous ceftriaxone plus Azithro or clarithromycin	SoC+; P	moderate to critical
66.	Johnston 2021	USA	231	1. HydroxyChl + Azithro N=77;<br>2. HydroxyChl N=71;	3. Placebo-equivalent (ascorbic acid and folic acid) N=83	No SoC	A+; P	moderate
67.	Joong 2021	ROK	307	1. CT-P59 40 mg/kg N=101;<br>2. CT-P59 80 mg/kg N=103;	3.Placebo N=103	No SoC	P	mild-to-moderate
68.	Kalil 2020	USA	1033	1. Remdes +Baricitinib N=515;	2. Placebo+Remdes N=518 SC	No SoC	A+; P	mild
69.	Kamran 2020	PK	500	HydroxyChl + SoC N=349;	2.SoC N=151	Vitamin C, vitamin D,zinc and tablet Paracetamol	SoC+	mild
70.	Khamis 2020	OM	89	1.Favipiravir + Interferon β 1b N=44;	2. HydroxyChl N= 44	Standard care based on the national guidelines that had HydroxyChl	SoC+	moderate/severe
71.	Kirti 2021	IN	115	1. Ivermectin + SoC N=55;	2.Placebo N=57 +SoC	No details on SoC reported	SoC-; P	mild-to-moderate

72.	Krolewicki 2020	RA	45	1. Ivermectin N=30;	2. SoC N=15	No details on SoC reported	SoC-	mild-to-moderate
73.	Kumar 2020	IN	32	1. Itolizumab + SoC N= 22;	2. SoC N=10	O2, antibiotics, HydroxyChl, antivirals, steroids, low molecular weight heparin and vitamins	SoC+	severe
74.	Lanzoni 2021	USA	24	1. Umbilical cord mesenchymal stem cell (UCMSC) infusions + SoC N=12;	2. SoC N=12	Best standard of care was provided in both groups following the current institutional COVID-19 guidelines.	SoC-	NR
75.	Lemos 2020	BR	20	1. Enoxaparin N= 10;	standard anticoagulant thromboprophylaxis N=10	No details on SoC reported	SoC-	severe
76.	Lenze 2020	USA	152	1. Fluvoxamine N=80;	2. Placebo N=72	No SC	P	NR
77.	Lescure 2021	RA, BR, CA, RCH, FR, DE, IS, I, JA, RU, E	416	1. Sarilumab 400 mg +SoC N=173;<br>2. Sarilumab 200 mg +SoC N=159;	3. Placebo +SoC N=84	Clarithromycin or Azithro; carbapenem; Oseltamivir; Lop/Rit; anticoagulants; corticosteroids (Methylprednisolone or Dexamethasone)	SoC+; P	severe
78.	Li C 2021	CN	94	1. genetically engineered recombinant super-compound interferon (rSIFN-co) + SoC N=46;	2. traditional interferon-alpha + SoC N=48	Lop/Rit or umifenovir	SoC+	moderate-to-severe
79.	Li L 2020	CN	103	1. Plasma C N=52;	2. SoC N=51	Support assistance for COVID-19, based primarily on China's evolving national COVID-19 treatment guidelines and hospital practice. Possible treatments include antiviral drugs, antibacterial drugs,	SoC+	Severe/critical

						steroids, human immunoglobulins, Chinese herbal medicines and other drugs.		
80.	Li T 2020	CN	18	1. Bromhexine Hydrochloride N=12;	2. SoC N=6	Antiviral drugs, including arbidol hydrochloride granules (0.1g-73 0.2g tid) and recombinant human interferon α2b spray (0.083ml tid),	SoC+	mild-to-moderate
81.	Li Y 2020	CN	86	1. Lop/Rit (200mg) + SoC N=34;	2. Umifenovir (100mg) + SoC N=35;<br>3. SoC N=17	support assistance and O2 therapy	SoC-	mild-to-moderate
82.	Libster 2020	RA	160	1. Plasma C N=80;	2. Placebo N=80	No SoC	P	mild
83.	Liesenborghs 2020	B	65	1. Itraconazole N=32;	2.SoC N=33	No details on SoC reported	SoC-	NR
84.	Lopes 2020	BR	75	1. Colchicine + SoC N= 37;	2. Placebo + SoC N=38	500 mg Azithro, 400 mg HydroxyChl twice daily for 2 days, then 400 mg once daily for 8 days, and unfractionated 5000 IU heparin three times daily until the end of hospitalization. Methylprednisolone 0.5 mg / kg / day for 5 days could be added if the need for supplemental O2 was 6 L / min or more	SoC+	moderate/severe
85.	Lopez-Medina 2021	CO	473	1. Ivermectin 300 µg/kg per day for 5 days N = 275;	2. Placebo N=198	No SoC	P	moderate

86.	Lou 2020	CN	30	1. Baloxavir + existing antiviral treatment N=10;	2: Favipiravir + existing antiviral treatment N=9;<br>3. existing antiviral treatment N=10	Lop/Rit (400mg / 100mg, twice a day) or darunavir / cobicystate (800mg / 150mg, four times a day) and Umifenovir (200mg, three times a day). All in combination with inhaled interferon alpha (100,000 iu, tid or qid).	SoC+	moderate/severe/critical
87.	Maldonado 2020	MEX	54	1. Pentoxifylline N=36;	2.SoC N=18	No details on SoC reported	SoC-	NR
88.	Mansour 2020	BR	30	1. Icatibant + SoC N=10;<br>2. Inhibitor of C1 esterase/kallikrein + SoC N=10	3.SoC N=10	Upon medical decision, patients received antibiotics, antithrombotic therapy, O2 support, non-invasive and invasive mechanical ventilation, vasopressor drugs, stress doses of corticosteroids and renal support therapy	SoC+	severe
89.	Mehboob 2020	PK	18	1. Aprepitant +dexamethasone N=8;	2. dexamethasone N=10	No SoC	A+	moderate/sevre/critical
90.	Miller 2020	USA	30	1. Auxora (low supplemental O2 flow) + SoC N= 17;<br>2. Auxora (high supplemental O2 flow) + SoC N=3;	3. SoC N=9	Antiviral agents, but investigational therapies and immunosuppressive medications were not permitted. At the discretion of the site investigators, patients treated with either Auxora or standard of care alone were able to receive Plasma C if they required invasive mechanical ventilation.	SoC+	severe
91.	Mitjà 2020	E	293	1. HydroxyChl+ SoC N=136;	2. SoC N=157	No details on SoC reported	SoC-	mild

92.	Mobarak 2021	IR	1083	1. Sofosbuvir + Daclatasvir + SoC N= 541;	2. Placebo + SoC N= 542	No details on SoC reported	SoC-	moderate/severe
93.	Mohan 2021	IN	125	1. Ivermectin 24 mg single dose + SoC N= 40;<br>2. Ivermectin 12 mg single dose + SoC n=40;	3. Placebo+SoC n=45	No details on SoC reported	SoC-; P	moderate
94.	Monk 2020	UK	101	1. Interferon beta 1a N=50;	2.Placebo+SoC N= 51	No details on SoC reported	SoC-; P	mild/moderate/sevre
95.	Morteza 2020	IR	180	1. Ivermectin (1 dose 200mcg/kg) N=30;<br>2. Ivermectin (3 doses 200mcg/kg) N=30;<br>3. Ivermectin (single dose 400mcg/kg) N=30;<br>4. Ivermectina (3 doses 400, 200, 200 mcg/kg) N=30	5. SoC N=30;<br>6. SoC+Placebo N=30	No details on SoC reported	SoC-; P	mild/moderate/sevre
96.	Nojomi 2020	IR	100	1. Umifenovir plus HydroxyChl N= 50;	2. Lop/Rit + HydroxyChl N=50	No SoC	A+	moderate/severe
97.	Omrani 2020	Q	456	1. HydroxyChl + Azithro N= 152;<br>2. HydroxyChl N= 152;	3. Placebo N= 152	No SoC	A+; P	mild/asymptomatic
98.	Rahmani 2020	IR	80	1. Interferon β-1b + SoC N=40;	2. SoC N=40	Lop/Ritr (400/100 mg BD) or atazanavir/ritonavir (300/100 mg daily) plus HydroxyChl (400 mg BD in first day and then 200 mg BD) for7–10 days.<br>Other supportive cares such as fluid therapy, stress ulcer prophylaxis,	SoC+	NR

						deep vein thrombosis, treatment of electrolyte disorders and antibiotic therapy were considered according to the hospital protocols.		
99.	Ramakrishnan 2021	UK	146	1. Inhaled budesonide N=73;	2. SoC N=73	paracetamol, or nonsteroidal anti-inflammatories such as aspirin and ibuprofen) and honey for symptoms of cough.	SoC+	mild
100.	Ranjbar 2021	IR	86	1. Methylprednisolone (2mg/kg/day) + SoC N= 44;	2.Dexamethasone (6mg/kg/day) + SoC N=42	No details on SoC reported	SoC-; A+	moderate/sevrere
101.	Rashad 2021	EG	306	1. Azithro 500 mg/24 h for 7 days N=107;	2. Clarithromycin 500 /12 h for 7 days N=99;<br>3. SoC N=99	Symptomatic treatment for control of fever and cough	SoC+; A+	NR

102.	Ray 2020	IN	80	1. Plasma C N=40;	2. SoC N=40	HydroxyChl, Azithro, Ivermectin and Doxycyclin; O2 therapy as per requirement; corticosteroids; therapeutic anticoagulation using either low molecular weight heparin or unfractionated heparin; appropriate broad-spectrum antibiotic therapy reservoir, high flow nasal cannula (HFNC) or in some cases mechanical ventilation (MV) were deployed. 1 patient in the SOC arm received Tocilizumab, none in the CPT arm. 13 patients in the SOC arm and 11 patients in the CPT arm received Remdes.	SoC+	severe
103.	RECOVERY Trial<br>Horby 2020<br>Horby 2020<br>Horby 2020<br>Horby 2020<br>Horby 2021	UK	4716<br>6425<br>5040<br>7764<br>4116	1. Hydroxych +SoC N015611561<br>1. Dexamethasone +SOC N= 2104;<br>1. Lop/Rit +SoC N= 1616;<br>1. Azythro + SoC N= 5182<br>1. Tocilizumab +SoC N= 2022	2. SoC N=3155;<br>2. SoC N= 4321;<br>2. SoC N= 1616;<br>2. SoC N= 5182<br>2. SoC N= 2094	No details on SoC reported	SoC-; A+	moderate/severe

104.	REMAP CAP Angus 2020 Gordon 2020	Europe; AU; NZ	403 826	1. Hydrocortisone (fixed dose 50 / 100mg every 6 hours) + SoC N=143;<br><br>1. Tocilizumab N= 353;	2. Hydrocortisone at the time of shock 50 / 100mg every 6 hours) +SoC N=152;<br>3. No hydrcortisone + SoC N=108<br><br>2. Sarilumab N=48;<br>3. SoC N=402	No details on SoC reported, the majority of patients (80%) glucocorticoids	SoC-; A+	
105.	Ren 2020	CN	20	1. Azvudina+ SoC N=10;	2. SoC N=10	Standard antiviral drugs: interferon alpha, kaletra + ribavirin + interferon alpha; kaletra + ribavirin; Chloroq and HydroxyChl. Symptomatic treatment: antibiotic therapy and traditional Chinese medicine	SoC+	mild/moderate
106.	Rocco 2020	BR	475	1. Nitazoxanide N=238;	2. Placebo N= 237	No SoC	P	mild/moderate
107.	Rosas 2020	CA, DK, FR, DE, IT, NL, E, UK USA	452	1.Tocilizumab (8 mg/kg infusion, maximum 800 mg) +SoC N= 301;	2. Placebo + SoC N= 151	Antiviral treatment, low-dose steroids, Plasma C, supportive care.	SoC+; P	severe
108.	Ruzhentsova 2020	RUS	168	1. Favipiravir (1800 mg BID on day 1, followed by 800 mg BID for up to 9 days) N= 112;	2. SoC N= 56	Umifenovir + intranasal interferon alpha-2b, or HydroxyChl for up to 10 days	SoC+	mild/moderate
109.	Sadeghi 2020	IR	70	1. Sofosbuvir/daclatasvir (400mg/60mg) N=35;	2. SoC N= 35	HydroxyChl 200mg twice daily with or without Lop/Ritr 200mg/50mg twice daily.	SoC+	moderate/sevre

110.	Sakoulas 2020	USA	34	1. Intravenous Immunoglobulin with 40 mg methylprednisolone N=17;	2. SoC N=17	Glucocorticoids, Plasma C, and Remdes or other treatments not included in the trial.	SoC+	moderate/severe
111.	Salama 2020	USA, MEX, EAK, ZA, PE, BR	388	1.Tocilizumab (IV 8mg/kg massimo 800mg+SoC N=259;	2. Placebo+SoC N=129	No details on SoC reported	SoC-; P	severe
112.	Salvarani 2020	I	126	1. Tocilizumab N=60;	2. SoC N= 66	Supportive care following the treatment protocols of each center including HydroxyChl, heparin and LMWH, antiretrovirals (darunavir/cobicistat, darunavir/ritonavir, or Lop/Ritr; Azithro	SoC+	severe
113.	Sekhavati 2020	IR	111	1. Azythro + Lop/Rit + HydroxyChl N=56;	2. Lop/Rit + HydroxyChl N=55	No SC	A+	NR
114.	Self 2020	USA	479	1. HydroxyChl + SoC N=242;	2. Placebo + SoC N=237	During Hospitalization: antivirals (open label HydroxyChl, Remdes, others); immunomodulator (corticosteroids, tocilizumab, other); antibacterial (Azithro); Plasma C	SoC+; P	severe
115.	Shi 2020	CN	101	1. Umbilical cord_ mesenchymal stem cells (hUC-MSC) + SoC N=66;	Placebo + SoC N=35	No details on SoC reported	SoC-	severe

116.	Shu 2020	CN	41	1. Umbilical cord_ mesenchymal stem cells (hUC-MSC) + SoC N=12;	2. SoC N=29	O2; antiviral agents (abidor/oseltamivir), antibiotic agents (moxifloxacin is taken orally; glucocorticoid therapy (1–2 mg/kg, less than a week).	SoC+	mild/moderate/severe
117.	Simonovic 2020	RA	334	1. Plasma C N=228;	2. SoC N=106	Antiviral agents, glucocorticoids, or both	SoC+; P	severe
118.	Soin 2021	IN	180	1. Tocilizumab + SoC N= 90;	2. SoC N= 90	Remdes + corticosteroids	SoC+	moderate/severe
119.	Solaymani-Dodaran 2021	IR	380	1. Favipiravir (1.6 gr loading, 1.8 gr daily) + SoC N= 193;	2. Lop/Rit (800/200 mg daily) + SoC N= 187	HydroxyChl 200 mg twice a day for 1 week	SoC+; A+	moderate/severe
120.	Spinner 2020	USA, Europe, Asia.	596	1. Remdes 5 days +SoC N=197;<br>2. Remdes 10 days +SoC N=199;	3 SoC N=200	No details on SoC reported	SoC-	moderate
121.	Stone 2020	USA	243	1. Tocilizumab +SoC N=161;	2.Placebo+SoC N=82	No details on SoC reported	SoC-; P	moderate/severe
122.	Tabarsi 2020	IR	84	Immunoglobulin 400 mg/Kg daily for three doses. Premedication: 500 mg Acetaminophen, 100 mg Hydrocort, and 25 mg Diphenhydramine 30 min before the injection N=52;	SoC N=32	O2 and fluid support, Lop/Ritr (200/50 mg, Hetero labs), two tablets twice a day, and HydroxyChl (Tehran-Daru) 200 mg two times daily.	SoC+	severe
123.	Tang W 2020	CN	150	HydroxyChl (1200mg for 3 days, followed by 800mg) +SoC N=75;	SoC N=75	Lop/Ritr, oseltamivir, entecavir, antibiotics, glucocorticoid	SoC+	mild/moderate/severe

124.	Tang X 2021	CN	86	1. Methylprednisolone + SoC N=43;	2. No Methylprednisolone + SoC N=43	standard therapy of COVID-19 according to the Chinese Diagnosis and Treatment Plan for COVID-19 (trial version 6)	SoC-	moderate/severe
125.	Tardiff 2021	CA	4488	1. Colchicine N=2235;	2.Placebo N=2253	No SoC	P	mild
126.	Tolouian 2021	IR	100	1. Bromhexine 8 mg four times a day + SoC N= 48;	2. SoC N=52	Lop/Ritr and interferon beta-1a	SoC+	moderate/severe
127.	Tomazini 2020	BR	299	1. Dexamethasone (20mg intravenously once daily for 5 days, followed by 10 mg intravenously once daily for additional 5 days or until ICU discharge, whichever occurred first N=151;	2. SoC N=148	No details on SoC reported	SoC-	critical
128.	Tornling 2021	SE	106	1. Oral angiotensin II type 2 receptor agonist C21 + SoC N=51;	2. Placebo+SoC N=55	antibiotics and antiviral compounds (Remdes in 67.0%), with no major differences between the treatment groups	SoC+; P	moderate
129.	Udwadia 2020	IN	150	1. Favipiravir +SoC N=75;	2. SoC N=75	Antipyretics, cough suppressants, antibiotics, and vitamins	SoC+	mild/moderate
130.	Ulrich 2020	USA	128	1. HydroxyChl + SoC N=67;	2. Placebo + SoC N= 61	No details on SoC reported	SoC-; P	mild/moderate/critical
131.	Veiga 2021	BR	129	1. Tocilizumab + SoC N=65;	2. SoC N=64	HydroxyChl, Azithro, corticosteroids, and antibiotics	SoC+	severe or critical
132.	Vlaar 2020	NL	30	1. Anti-C5a antibody (IFX-1) + SoC N=15;	2. SoCN=15	No details on SoC reported	SoC-	moderate/severe/critical
133.	Wang 2020	CN	237	1. Remdes + SoC N=158;	2. Placebo+SoC N=79	Lop/Rit; Antibiotics; Corticosteroids	SoC+; P	severe

134.	Wang D 2020	CN	65	1. Tocilizumab + SoC N=33;	2. SoC N=32	No details on SoC reported	SoC-	moderate/severe
135.	Wang M 2020	CN	50	1. Leflunomide plus Interferon α-2a N= 26;	2. Interferon α-2a N= 24	No SoC	A+	NR
136.	Weinreich 2021	USA	269	1. REGN-COV2 at a dose of 2.4 g N=92;<br>2, REGN-COV2 at a dose of 8.0 g N=90;	3.Placebo N=93	No SoC	P	mild
137.	WHO-Solidarity trial	WHO countries Solidarity trial Consortium	14256	1. Remdes +SoC N= 2750;<br>1. HydroxyChl + SoC N=954;<br>1. Lop/Rit + SoC N=1411;<br>1. Interferon β-1a + SoC N=2063:	2. SoC N=2725<br>2. SoC N=909<br>2. SoC N=1380<br>2. SoC N=2064	No details on SoC reported	SoC-; A+	mild/moderate/severe
138.	Wu 2020	CN	52	1. Triazavirin (moderate: 250 mg orally 3 times / day; severe / critical: 250 mg orally 4 / day) + SoC N=26;	2.Placebo + SoC N= 26	Antibiotics, Antiviral drugs, Interferon, Arbidol, Ribavirin, Lopinavir, HydroxyChl, Antifungal drugs, Chinese medicine therapy	SoC+; P	moderate/severe/critical
139.	Yadollahzadeh 2021	IR	112	1. Sofosbuvir + Daclatasvir N= 58;	2. Lop/Rit N= 54	No SoC	A+	moderate
140.	Yakoot 2020	EG	89	1. Sofosbuvir plus Daclastavir + SoC N=44;	2. SoC N=45	Standard Care as per the Egyptian Ministry of Health (MOH) protocol: HydroxyChl multivitamin and zinc supplements with vitamin C and D. Other drugs were given according to each patient clinical condition and included: analgesic-antipyretic, cough mixtures, Parenteral antibiotics, heparin/low	SoC+	mild, moderate, severe

						molecular weight heparin/ or novel oral anticoagulants, conservative IV fluid management if indicated and O2 therapy, up to mechanical ventilation as needed.		
141.	Youssef 2021	USA	196	1. Aviptadil (Vasoactive Intestinal Peptide) +SoC N= 131 vs	2. Placebo+ SoC N=65	No details on SoC reported	SoC-	moderate
142.	Zarychanski-2021	CA	108 9	1. Heparin + SoC N=532;	2. Pharmacological thromboprophy laxis + SoC N=557	No details on SoC reported	SoC-	severe
143.	Zhao 2020	CN	26	1. Favipiravir (1600mg day 1 then 600mg) + Tocilizumab (4-8mg/kg) N=14;	2. Favipiravir (1600mg day 1 then 600mg) N=7;<br>3. Tocilizumab (4-8mg/kg) N=5	No SoC	A+	moderate/se vere/critical
144.	Zheng 2020	CN	89	1. Novaferon (40 mg) N=30;	2. n=30 Novaferon (40 mg) + Lop/Rit (200/50mg) N=30;<br>3. Lop/Rit (200/50mg) N=29	No SoC	A+	moderate/se vere

HydroxyChloroquine= HydroxyChl; Lopinavir/ritonavir= Lop/Rit; Remdesivir= Remd; Convalescent plasma = Plasma C; Azithromycine= Azithro; Favipavir= Favi; Tocilizumab= Toci; Chloroquine= Chloroq; Colchicine= Colchi; Ivermectine= Iver; Ribavirine= Riba; Methylprednisolone= Methylpredni

Egypt: EG; Spain: ES; Francia: FR; China: CN; United States of America: USA; Islamic Republic of Iran: IR; Colombia: CO; Canada: CA; Australia: AT; Denmark: DK; Nigeria: NG; Pakistan: PK; Brazil: BR; Latino América: AR; Czech Republic: CZ; Italy: IT; Netherlands: NL; Belgium: BE; Sweden: SE; Norway: NO; Peru: PE; Vietnam: VN; India: IN; Tunisia: TN; United Kingdom: UK; Germany: DE; Hong Kong: HK; Japan: JP; Republic of Singapore: NL; Taiwan: TW; Korea: KR
